# Parapneumonic empyema in children: a scoping review of the literature

**DOI:** 10.1186/s13052-024-01701-1

**Published:** 2024-07-30

**Authors:** Danilo Buonsenso, Francesca Cusenza, Lucrezia Passadore, Francesca Bonanno, Carolina Calanca, Francesco Mariani, Carlotta Di Martino, Sonia Rasmi, Susanna Esposito

**Affiliations:** 1 Department of Woman and Child Health and Public Health, Fondazione Policlinico Universitario A. Gemelli IRCCS, Rome, Italy; 2 Pediatric Clinic, Department of Medicine and Surgery, University of Parma, Parma, Italy; 3 Medicine and Surgery, Catholic University of Rome, Rome, Italy

**Keywords:** Antibiotics, Children, Empyema, Pneumonia, Surgery

## Abstract

Community-acquired pneumonia can lead to a serious complication called empyema, which refers to pus within the pleural space. While it poses a significant threat to morbidity, particularly in children, it is fortunately not associated with high mortality rates. However, determining the best course of management for children, including decisions regarding antibiotic selection, administration methods, and treatment duration, remains a topic of ongoing debate. This scoping review aims to map the existing literature on empyema in children, including types of studies, microbiology, therapies (both antimicrobial and surgical) and patient outcomes. We systematically searched PubMed and SCOPUS using the terms “pediatric” (encompassing children aged 0 to 18 years) and “pleural empyema” to identify all relevant studies published since 2000. This search adhered to the Preferred Reporting Items for Systematic Reviews and Meta-Analyses extension for Scoping Reviews (PRISMA ScR) checklist.

A total of 127 studies was included. Overall, 15 attempted to compare medical treatments (alone or in combination with pleural drainage or fibrinolysis) with more invasive surgical approaches, and six studies compared diverse surgical interventions. However, the diversity of study designs makes it difficult to derive firm conclusions on the optimal approach to pediatric empyema. The heterogeneity in inclusion criteria, pharmacological/surgical approaches and settings limit the ability to draw definitive conclusions. Overall, 78 out of 10,896 children (0.7%) included in the review died, with mortality being higher in Asia and Africa. Our scoping review highlights important gaps regarding several aspects of empyema in children, including specific serotypes of the most common bacteria involved in the etiology, the optimal pharmacological and surgical approach, and the potential benefits of newer antibiotics with optimal lung penetration. New trials, designed on a multi-country level a higher number of patients and more rigorous inclusion criteria and designs, should be urgently funded.

**Supplementary Information:**

The online version contains supplementary material available at 10.1186/s13052-024-01701-1.

## Introduction

Parapneumonic empyema, characterized by pus accumulation on the pleura, is a common local complication of childhood community-acquired pneumonia (CAP) [1–5]. While estimates suggest parapneumonic effusions develop in approximately 1 in every 100 to 150 children with CAP [6, 7], hospitalized children with CAP may have a prevalence as high as 40% [8].

The primary causative agent of CAP is predominantly *Streptococcus pneumoniae*, and its incidence has demonstrated fluctuations over time [9]. Notably, there has been a significant global decrease in pneumococcal disease and mortality rates following the introduction of the heptavalent pneumococcal conjugate vaccine (PCV7), which targets serotypes 4, 6B, 9 V, 14, 18 C, 19 F, and 23 F, into routine childhood immunization schedules [10]. However, in subsequent years, the USA observed an uptick in pneumococcal empyema cases associated with serotypes not covered by PCV7 [11]. Following the transition from PCV7 to PCV13, which additionally includes pneumococcal serotypes 1, 3, 5, 6 A, 7 F, and 19 A, there has been a noteworthy reduction in the incidence and hospitalization rates related to empyema [12]. This shift to PCV13 is particularly significant given the strong correlation between parapneumonic empyema and pneumococcal serotype 1 [13]. Although other bacteria, such as group A *Streptococcus* and *Staphylococcus aureus*, are less commonly linked with CAP, they are potential bacterial pathogens associated with parapneumonic empyema [13].

The clinical manifestation of parapneumonic empyema closely resembles that of uncomplicated CAP [9, 13]. Suspecting empyema is prudent in children experiencing prolonged fever (lasting 7 days or more) or those showing no improvement after 48–72 h of appropriate antibiotic therapy. Physical examination typically reveals reduced air entry and dullness to percussion [9]. Chest X-ray and/or pulmonary ultrasound are used to confirm suspected parapneumonic empyema. Ultrasound is particularly valuable due to its higher sensitivity compared to X-ray in assessing fluid collection extension and nature; additionally, it avoids radiation exposure for children. While thoracic computed tomography (CT) isn’t a first-line diagnostic tool for empyema, it may be warranted when diagnosis is unclear or malignancies are suspected, such as Burkitt’s lymphoma.

Treatment for parapneumonic empyema always includes empiric intravenous broad-spectrum antibiotic therapy targeting common bacteria like *Streptococcus pneumoniae*,* Streptococcus pyogenes*, and *Staphylococcus aureus* [5, 14]. In cases of significant effusion (> 2 cm) or respiratory compromise, chest drainage is recommended [14]. Ultrasound-guided chest drainage is standard, often performed with children under sedation or general anesthesia. Intrapleural fibrinolytics, like urokinase, can expedite hospital discharge for cases with slow drainage or thick, loculated fluid [5, 14]. Thoracic surgery should be considered in cases of antibiotic therapy failure, ineffective chest drainage, or inadequate response to fibrinolytics. However, current guidelines lack clear recommendations on the ideal surgical procedure, timing of intervention, duration of drainage and antibiotic therapy, transition to oral antibiotics, and how these factors influence outcomes. This scoping review aims to comprehensively outline the literature on study types, microbiology, therapeutic interventions (both antimicrobial and surgical), and outcomes of empyema in children since 2000.

## Methods

### Review questions

To address the lack of consensus on optimal treatment for pediatric parapneumonic empyema literature [15], this review primary focus will be to examine the existing literature on antibiotic and surgical interventions about pediatric empyema. This will include investigating the selection of first-line agents, appropriate dosages, routes of administration, and treatment durations. Furthermore, this review will address the following sub-questions:

1. What are the most commonly identified pathogens reported in literature?
2. What are the predominant outcomes and complication rates associated with empyema, as reported in the literature?
3. Which conservative or invasive treatments are most frequently reported, and which demonstrate improvements in outcomes and reduced length of stay?

The protocol for this review has been published prospectively and can be accessed at https://osf.io/9wkma/.

### Inclusion criteria

This review encompasses studies involving children and adolescents (under 18 years old) who have received a confirmed diagnosis of empyema, defined by the presence of pus within the pleural cavity. Diagnosis of empyema is established through the identification of pus, positive Gram’s stain, culture, or nucleic-acid amplification tests in the pleural fluid. Only studies explicitly mentioning the performance of microbiological investigations, administration of antimicrobial and surgical treatments, as well as outcomes (at least until discharge), have been included.

The primary focus of this review is to comprehensively examine all aspects of empyema, with particular attention given to treatment options. Due to the severity of the condition, articles involving non-hospitalized patients were not anticipated, thus only inpatient studies have been considered.

To capture a broad range of evidence, this review includes randomized controlled trials, non-randomized controlled trials and all observational studies, (prospective and retrospective, including case-control, cohort, and cross-sectional studies, as well as small case series or single case reports).

### Search strategy

The search was conducted by one reviewer. It began in April 2023, using the bibliographic databases PubMed and SCOPUS. We limited our search to English-language articles published between January 1, 2000, to March 31, 2023. The search strategy incorporated a combination of keywords and their synonyms, including “pediatric,” “empyema,” and “treatment.” The PubMed search strategy is accessible in the supplementary data section of this protocol; the terms used in this search were adjusted for use with other bibliographic databases.

Following the search, studies were exported to Rayyan. Initially, one author screened for duplicates. Subsequently, titles and/or abstracts of retrieved studies were independently screened by two reviewers to identify potentially relevant studies for inclusion in the review. Full texts of potentially eligible studies were then retrieved and independently assessed for eligibility by two reviewers. Each researcher was blinded to the decision of the other. Any discrepancies regarding study eligibility were resolved through discussion and, if necessary, consultation with a third reviewer.

Studies failing to meet the inclusion criteria were excluded, and a table detailing the reasons for exclusion was included in the final manuscript. The results of the search were reported using the PRISMA flow diagram.

Data extraction was performed independently by two review authors, each using a separate Excel spreadsheet. Each researcher remained blinded to the other’s decisions. In cases of discordance, disagreements were identified and resolved through discussion (with involvement of a third author if needed).

An Excel file was utilized to store extracted data, which included the following when available:

1. Study details: title, author, year of publication, study type, number of patients, geographic location.
2. Participant characteristics: sample size, nationality, age, socio-economic status, comorbidities.
3. Clinical manifestations: fever duration, cough with mucus, dyspnea, chest pain, and others.
4. Imaging findings: lung involvement type on chest X-rays, lung ultrasound (US), CT scans, or MRI.
5. Details of antimicrobial treatments administered during empyema (e.g., duration, antibiotics used).
6. Adjunctive treatments and their durations during empyema (e.g., steroids, other immunomodulatory medications).
7. Surgical interventions and their durations during empyema (e.g., drainage, thoracoscopy, surgical resection).
8. Outcomes (e.g., death, survival, survival with sequelae, type of sequelae).

### Data analysis and presentation

To present our findings, we adhered to the Preferred Reporting Items for Systematic Reviews and Meta-Analyses extension for Scoping Reviews (PRISMA-ScR) Checklist, as detailed in the supplementary material. A narrative synthesis was conducted to summarize the results obtained from the studies included in the review, providing our interpretation of the findings. Special attention was given to antimicrobial and surgical therapies, with a focus on the frequency of antibiotic selection, efficacy, and treatment duration. more than 100 records were included after the initial selection process, preference was given to original articles and those published within the last 5 years.

Tables and charts were employed to concisely summarize both the characteristics of included studies and essential clinical, diagnostic, treatment, and outcome data. Various tables and figures were compiled to outline the types of studies and their primary findings, covering microbiology, therapies, and outcomes. Additionally, we emphasized areas for future research to address existing gaps in knowledge.

### Patient and public involvement

Patient and public involvement was not directly incorporated into this review. However, the primary inquiries that motivated our research project were influenced by public dialogues initiated by family associations in the media. These conversations underscored the significance of gaining a deeper understanding of how empyema can be identified earlier in the disease progression, prior to clinical deterioration becoming uncontrollable. Additionally, they raised questions about the potential for preventing empyema if it arises as a complication of a previously undetected and untreated lung infection.

## Results

### Characteristics of available literature

**Fig. 1:**
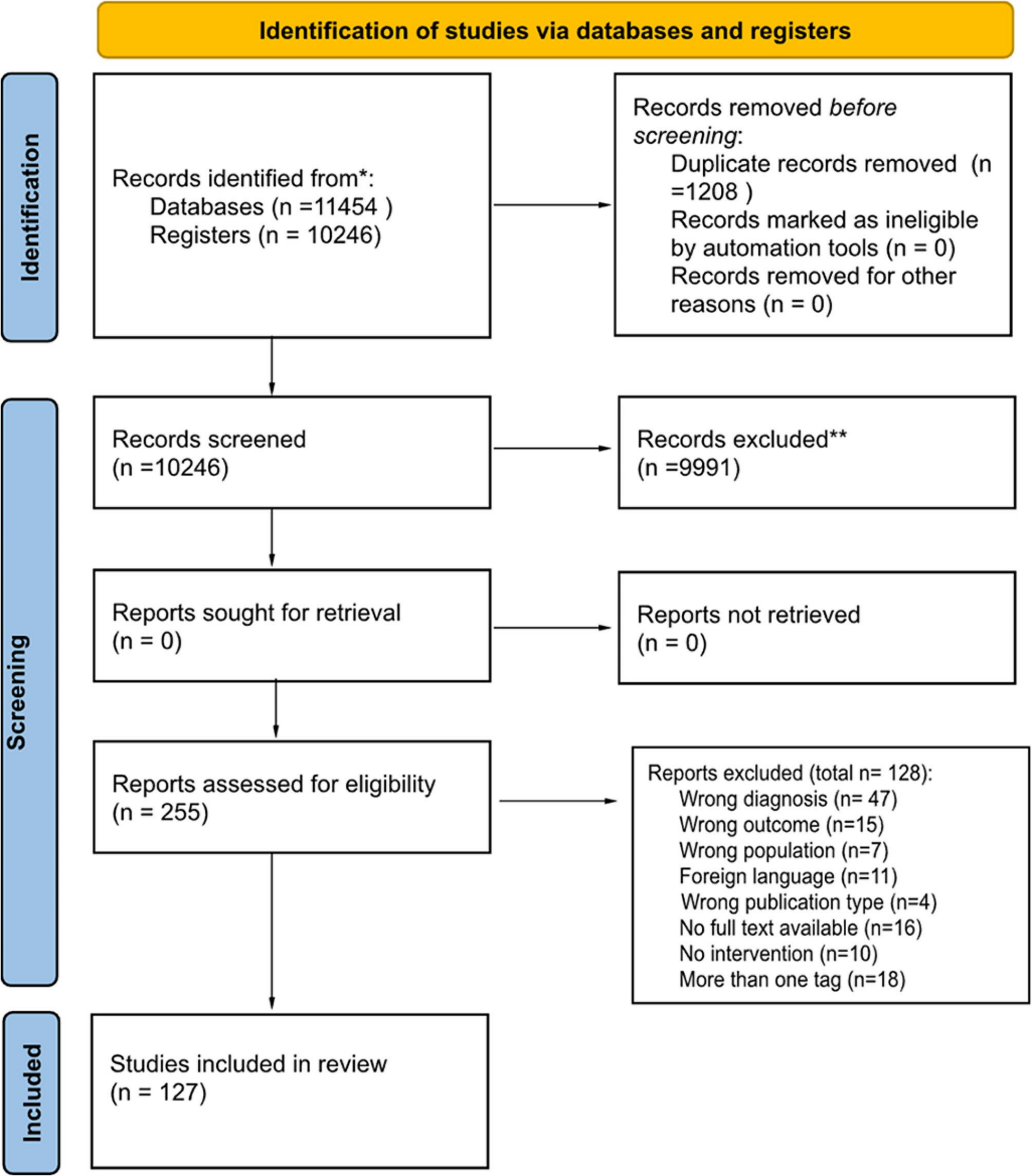
PRISMA flowchart of included studies

**Fig. 2:**
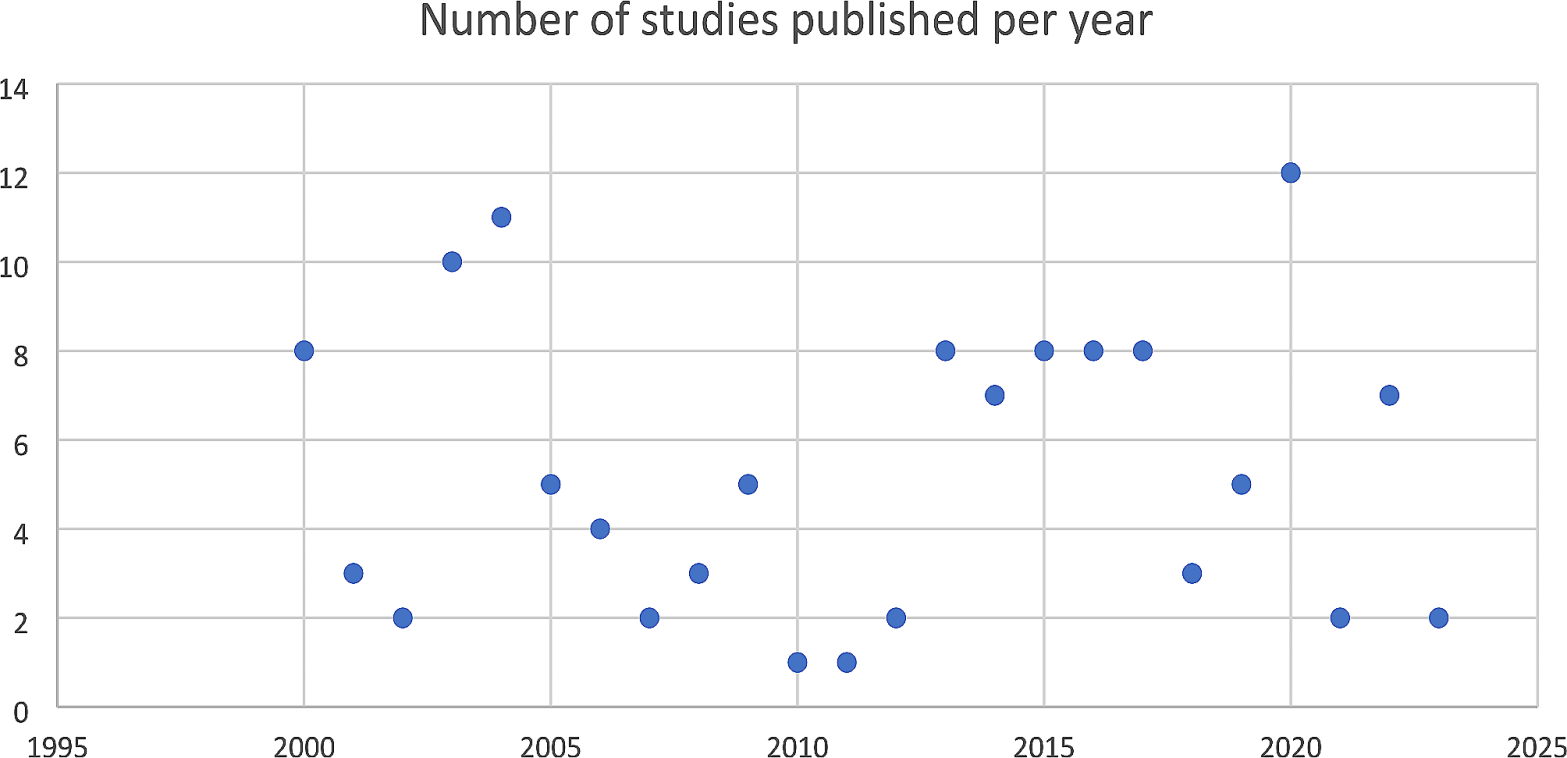
Temporal distribution of studies on pediatric empyema

We reviewed literature published between January 2000 to January 2023 and identified 127 articles in our systematic review. Figure 1 shows the included studies according to PRISMA flowchart. The majority of studies were observational studies (93), 77 retrospective and 16 prospective, 20 case series, 9 randomized clinical trials and 5 case series. Geographically, most studies originated from Asia, Europe and North America, with a minority from low-income countries. Interestingly, Fig. 2 reveals a slight increase in publications on pediatric empyema over the last decade.

### Microbiology of empyema

All 127 studies in our scoping review assessed microbiological etiology of empyema. Microbiological examinations were performed on either pleural fluid or biological samples obtained from bronchoalveolar lavage, blood culture, or a combination of two or more of these samples. Overall, 2% (*n* = 2) of the studies, despite investigating the microbiological etiology, did not provide quantitative results or the percentage of patients for each pathogen. Another 2% (*n* = 2) of the studies did not report microbiological results in the specific text. Moreover, 6% (*n* = 9) of the studies reported that the examined microbiological samples yielded negative results. Finally, 90% (*n* = 114) of the studies specified the pathogen by reporting the number of patients with positive results.

**Fig. 3:**
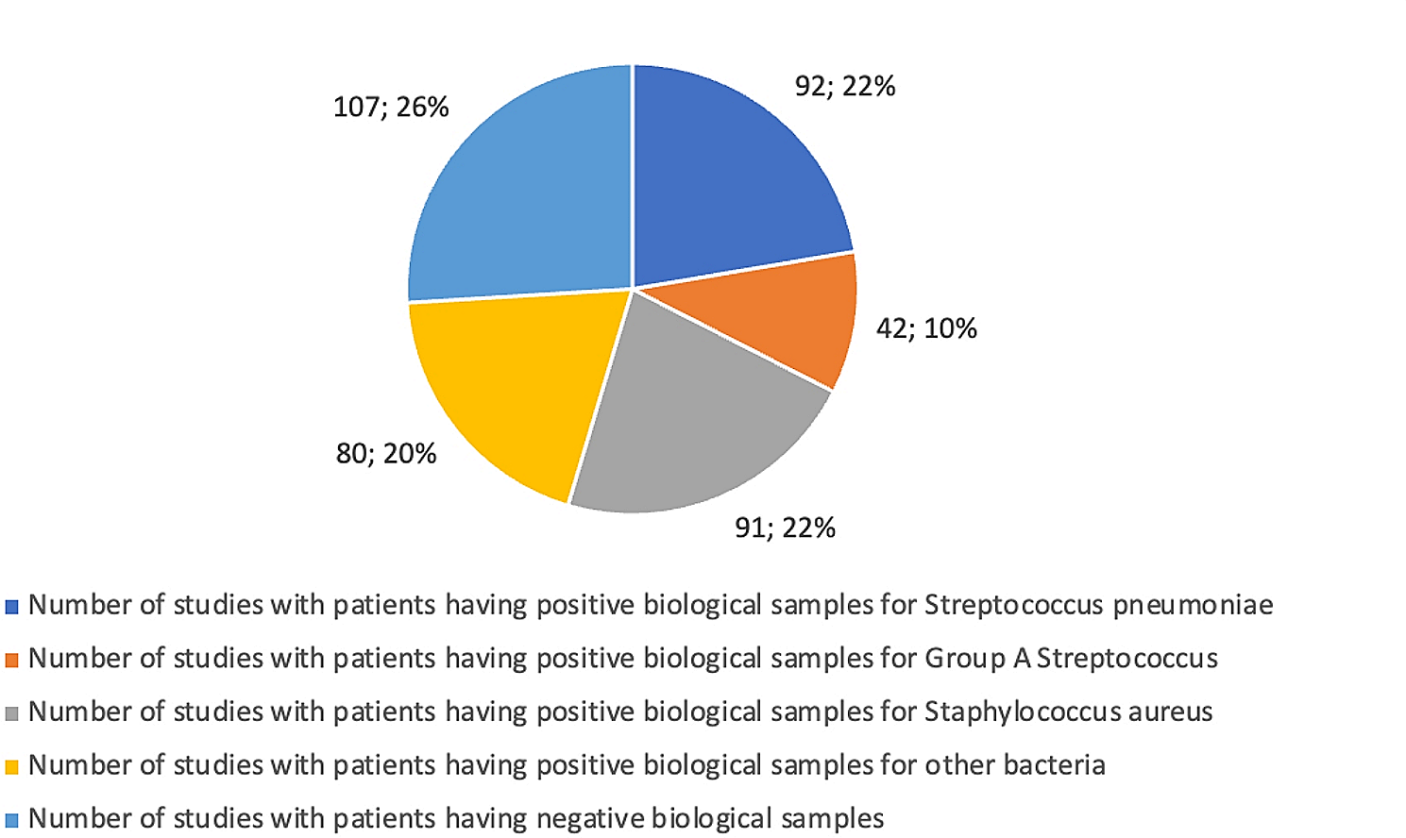
Description of bacteria involved in the pathogenesis of empyema

In the 114 studies where the microbiological diagnosis of empyema was specified, various etiological agents were implicated. *Streptococcus pneumoniae* was highlighted in patients from 92 studies, group A *Streptococcus* in 42, and *Staphylococcus aureus* in 91 (Fig. 3). In 80 studies, other microorganisms implicated were reported, different from those previously mentioned, including: *Haemophilus influenzae*, *Streptococcus anginosus*, *Streptococcus viridans*, *Escherichia coli*, *Neisseria spp*, *Klebsiella pneumoniae*, *Pseudomonas aeruginosa*, *Fusobacterium necrophorum*, coagulase-negative *Staphylococcus*, *Enterococcus* spp., *Enterobacter aerogenes*, *Enterobacter cloacae*, and influenza A, as well as fungi. Notably, 107 studies documented negative microbiological results.

### Antibiotic treatment

Details on medical therapy were available only in 71 out of the 127 included studies. In 30 cases out of 71, the molecule used was not specified. Of the studies reporting specific antibiotics, the rest of them was mentioned as follows, often administered in combination: in 73.1% (*n* = 30 studies) Ceftriaxone was the most frequent, followed by Vancomycin 46.3% (*n* = 19), Amoxicillin-Clavulanic acid, 41.4% (*n* = 17), Clindamycin 41.4% (*n* = 17), Linezolid 17% (*n* = 7) and Teicoplanin 4.8% (*n* = 2). Furthermore, no studies compared the effectiveness of different antibiotic classes, single vs. combination therapies, or explored variations in treatment durations and administration routes (fully intravenous vs. partial intravenous vs. fully oral).

### Surgical management

**Fig. 4:**
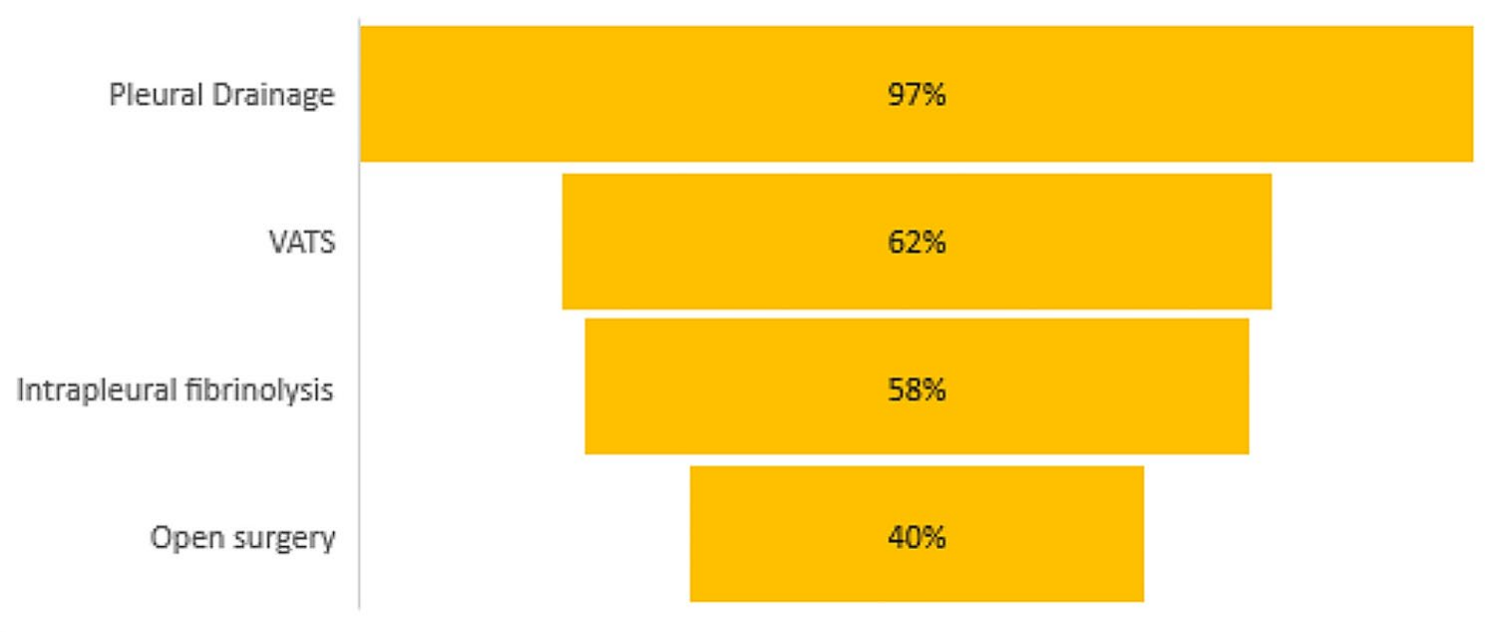
Surgical treatments performedin studies on pediatric empyema

The studies employed various surgical approaches, as detailed in Fig. 4. Pleural drainage was the most frequent procedure, reported in 123 studies (97%), intrapleural fibrinolysis in 73 (58%), video-assisted thoracoscopy surgery (VATS) in 79 (62%), surgical treatments as lung decortication or lobectomy in 51 (40%). In 26 studies (20%), all types of surgical techniques were performed, mostly in Europe and Asia, while in 18 (14%) only pleural drainage and in 3 (2%) only VATS was conducted. The remaining studies involved combinations of various surgical techniques. Most studies originated from Asia, Europe and North America, and the application of surgical techniques displayed significant geographical variability.

It’s noteworthy that VATS procedures became more prelevant from 2019 onwards, whereas their use was less consistent in earlier years. Similarly, the use of fibrinolysis primarily began after 2012.

### Treatment comparisons

Fifteen studies tried to compare medical treatments (alone or in combination with pleural drainage or fibrinolosis) with more invasive surgical approaches, and 6 studies that compared diverse surgical intervetions. Nearly half of these studies originated from Asia.

To assess the safest and most effective tratmente, these studies employed various criteria, which exhibited significant heterogeneity between studies.

Interestingly, most studies considered the total duration of hospitalization as a marker of treatment severity and, indirectly, treatment success.

However, the different inclusion criteria, diverse pharmacological/surgical approaches and study settings, do not allow to reach firm conclusions. Table 1 summarizes the main findings from those studies that attempted to perform specific therapeutic comparisons [16–37].

**Table 1:** Characteristics of studies that compared different interventions

First Author (year)	Region	Patients (*N*)	Intervention 1	Intervention 2	Lenght of stay	Outcome	Other observations
Evie Yeap (2019) [16]	Oceania	159	Broad spectrum intravenous antibiotic therapy	Broad spectrum intravenous antibiotic therapy + VATS	Significantly longer in the VATS group than in the no VATS group (*P* < 0.0001)	VATS was completed in all patients with no intraoperative complications. There were no significant perioperative complications or deaths.	- Patients requiring VATS were significantly younger than patients successfully treated with medical therapy alone (*P* = 0.004). - There was no difference in seasonal presentation between the 2 groups (*P* = 0.8) or the median duration of symptoms before hospitalization (*P* = 0.8). - Duration of oral antibiotic therapy after discharge was similar between the 2 groups
Patrick M. Meyer Sauteur (2019) [17]	Europe	147	Empirical treatment with AMX/AMC (100 mg/kg/day IV in 3 doses) or targeted treatment according to microbiological test results .	Empirical treatment with AMX/AMC (100 mg/kg/day IV in 3 doses) or targeted treatment according to microbiological test results combined with surgical intervention (chest tube drainage alone or with intrapleural fibrinolytic therapy, VATS and thoracotomy	PPE/PE patients with surgical interventions were significantly longer hospitalized than those with pleural tap alone (LOS 21 vs. 9 days, *p* < 0.001)	Five PPE/PE patients experienced poor outcome, defined as incomplete clinical and/or radiological recovery. Thereof, only two (1%) suffered from clinical impairment (chronic pneumopathy), while the three others reached complete clinical recovery. Although the final outcome was comparable between patients with surgical interventions and those with pleural tap alone (complete recovery in 95% vs. 98%, *p* = 0.401), patients that underwent surgical intervention needed significantly more time to recover. There was no difference in outcome of children with different methods of surgical interventions	Patients that underwent surgical intervention were more likely to have a positive pleural fluid Gram stain (*p* = 0.002), an increased white blood cell (WBC) count (*p* < 0.001), and a large size (*p* < 0.001) and organized (*p* = 0.011) effusion
Shyam Prasad Kafle (2022) [18]	Asia	106	Intercostal tube drainage and antibiotics with / without fibrinolytic therapy.	Antibiotics + both intercostal drainage and surgical management ( VATS and decortication)		Out of 106 children, three (2.84%) patients died during the treatment in the hospital. Eight patients developed broncho-pleural fistula. Out of these, six had broncho-pleural fistula at the time of admission. Of these, two recovered during the hospital stay, and four were asked to follow up in the CTVS OPD at the time of discharge. The overall success rate of medical management for empyema was 83.96%. Good pulmonary outcome was noted with conservative therapy alone	
Maria Rosa Ibarra Rodríguez (2022) [19]	Europe	35	Urokinase (UK) Fibrinolytic instillation was started after drain placement or the following day and consisted of 3 daily boluses of a 1000 intrapleural units (IU)/ml dilution of UK in saline at a dose of 10,000 IU/kg/day.	Thoracoscopy (TS)	The preprocedural stay was longer in the TS group (4 days, IQR 3.5, vs. 1.5 days, IQR 1; *P* < 0.001); the postprocedural stay was shorter (9 days, IQR: 7, vs. 12 days IQR: 5), close to significance (*P* = 0.09).	According to the treatment-related complications, none of patients suffered anaphylaxis after UK administration. Any bronchopleural fistula, prolonged air leak, or lung collapse was found after any of the procedures. One patient presented with post-TS bleeding with a decrease of more than two hemoglobin points requiring transfusion but not reoperation. None of the patients treated with TS required reconversion to TT. There were also no significant differences in the time to radiological normalization, which was 2 months in both groups.	The days of antibiotic therapy after the procedures were 8 days in the group who underwent thoracoscopy (TS) and 11 days in the group who underwent intrapleural instillation of fibrinolytic urokinase (UK) (*P* = 0.03).
Anil Gautam (2022) [20]	Oceania	153	Medical ( Antibiotics alone Antibiotics and ICC Fibrinolytics)	Surgical (VATS Thoracotomy)	Increased length of stay with nonoperative drainage procedures in the overall cohort (14 vs. 11.3 days, *P* = 0.07).	High failure rate with medical therapy (almost 50% requiring a repeat procedure on the ipsilateral side or in some isolated cases, delayed intervention after initial planned conservative approach). A significant majority of patients (82%) were transferred to the intensive care unit (ICU) at some stage in their hospital journey. ICU admission was to support acutely unwell children, both for monitoring pre/postprocedure as well as for procedural sedation and pigtail/chest drain insertion where surgery was not contemplated.	
Akın Eraslan Balcı (2002) [21]	Europe	71	Intrapleural fibrinolytic therapy. (urokinase instillation)	Operative group (decortication)	Post-intervention hospital stay was less in operative group (8.7 days) than fibrinolytic group (9,5 days).	Duration of initial chest tube was more in operated group (11.2 days) than fibrinolytic group (9.1 days). Occurred 1 death in fibrinolytic group.	After intervention, the mean empyema severity score of the Operated Group was less than that of the fibrinolytic group (*P* < 0,05).
Pwk Chan (2000) [22]	Europe	54	Chest tube and antibiotics	Surgical intervention with thoracotomy and decortication.	Patients who underwent surgery had a longer hospital stay of 18.6 ± 9.1 days compared with 13.4 ± 5.3 days in patients who received only medical treatment (*P* = 0.001).	All patients were discharged well after a mean hospital stay of 15.4 ± 7.4 days and median of 14.5 days. There were no deaths.	There was no difference in any of the initial clinical presenting features between those who did or did not have surgery, apart from later insertion of a chest drain at 8.1 ± 5.4 days after the appearance of the pleural effusion, compared to 6.3 ± 5.2 days in patients who showed good response to medical management alone. This difference failed to reach statistical significance (*P* = 0.57). Many of the patients in this series failed to respond to conservative treatment because of the inadequate size of the initial chest tube. Delayed and inadequate pus drainage in pleural empyema at the onset of illness will make recovery with medical management less likely and increases the need for thoracotomy and decortication.
KR Shankar (2000) [23]	Europe	47	Conservative treatment (antibiotics alone or in combination with tube thoracostomy)	Thoracotomy and debridement or decortication.	Three out of 15 patients treated non-operatively developed complications in the form of recurrent effusion and persistent fever requiring a prolonged hospital stay and antibiotic therapy.	Seven out of 32 children treated surgically experienced significant morbidity. These complications were seen in the children in whom thoracotomy was significantly delayed (*p* = 0.045. Their complications included: recurrent empyema with lung abscess, scoliosis, restric-tive lung disease, bronchopleural fistula and sympathetic pericardial effusion.	
K.D. Schultz (2004) [24]	North America	212	Non surgical group (antibiotics only, or had a thoracentesis and/or had a chest tube placed, )	Surgical group (some type of surgical intervention: VATS, minithoracotomy, or open thoracotomy)	LOS significantly shorter for patients who were treated with early VATS The 4-day reduction in hospitalization is clinically significant.	Complications, including lung abscess, pneumatocele formation, bronchopleural fistula formation, respiratory failure, requirement of a blood transfusion, and air leak 24 h, were not statistically significant between the nonsurgical and surgical groups. Eight patients required lobectomy during the study period. There were no deaths from empyema associated with community-acquired pneumonia during the study period.	Early VATS is an effective and safe method of treating empyema in children and should be considered a treatment of choice. Patients who underwent early VATS had a decreased length of fever overall. Most patients in the early VATS group were afebrile by day 5, 10% remained febrile on day 15, with 1 patient febrile on day 28. Therefore, persistently febrile patients should be watched closely with no additional intervention unless there is a change in the patient’s status.
E. Eroǧlu (2004) [25]	Asia	93	Conservative management Chest tube drainage and antibiotics If the patient had developed loculations and there was entrapment of the lung in an organized peel, then intrapleural urokinase (UK) administration was carried out.	Surgical intervention (decortication)	The mean hospitalization of 24 days for the conservative group, was similar to that of other reports Although the mean hospital stay for the decortication group was 11 days, which is shorter compared to the conservative group, these patients had previously been hospitalized in different institutions for an average period of 2 months. Thus, the average hospital stay for these patients was in reality much longer than for the conservative treatment group. After the removal of the inelastic pleura, the lung expanded and the patients recovered quickly.	In our series, 87.6% of the patients were treated successfully with a conservative approach. There was no mortality Medical management with adequate chest tube drainage and appropriate antibiotics resulted in full resolution in the majority of patients with empyema.	In cases of loculated pleural effusions identified during the early stages of chest tube drainage, intrapleural UK administration was found to be a safe and efficient treatment modality. Decortication, if performed with correct indications in suitable patients, speeds up patient recovery and should be reserved for late and organized empyema cases.
N. Kalfa (2004) [26]	Europe	20	Group 1 (early thoracoscopy) defined as those who underwent surgical procedures immediately after diagnosis or after a brief trial (3 days) of medical treatment (antibiotics and nonsurgical empyema evacuation). Group 1 then was divided further to compare the children who had undergone immediate VATS and those who initially had a brief trial of medical treatment.	Group 2 (later thoracoscopy) included children who underwent VATS after day 4.	First-line systematic VATS was compared with initial brief medical treatment followed by thoracoscopy before day 4. This first medical step did not seem to increase operative time, duration of drainage, or LOS	Complications were present only in group 2: two relapses of pneumothorax by air leakage from lung parenchyma with necrotizing pneumonia and one recurrence of empyema. Two additional VATS were required in this group. Comparison of group 1 children who had immediate VATS and those who had first-line brief medical treatment: There was no significant difference between these two subgroups, especially in the important prognostic criteria of operative time, the need for additional procedures, and the complications	Our study does not support using VATS as a systematic first-line therapy. Medical treatment will show its efficacy within the initial period of 4 days. Clinically, improvement in respiratory symptoms can be gauged by reduced dyspnea and effective fluid evacuation as confirmed by radiography. Fever, on the other hand, may persist. Waiting longer may affect prognosis negatively. Of course, if initial imagery shows pleural organization, VATS should be proposed as the initial treatment.
A.F. Saleem (2014) [27]	Asia	112	Antibiotics and supportive treatment only.	Managed surgically (chest drain only, chest drain and decortication, chest drain, decortication and pneumonectomy)	Children managed medically has shorter length of stay (*p* = < 0.001) compared to children managed surgically.	Children managed medically has less thrombocytosis (*p* = 0.06) compared to children managed surgically. Three patients died; 2 admitted with severe pneumonia and empyema and respiratory failure, found to be necrotizing pneumonitis and one with severe sepsis, and disseminated intravascular coagulation. Forty-one (37%) patients were discharged with chest drain, and total duration of chest drain was 12 ± 11 days. Eight patients were readmitted within 72 h of discharge. Fifteen patients developed complications (subcutaneous emphysema, recollection of pus, rib osteomyelitis, bronchopulmonary fistulae and pneumatocele) .	Surgically managed children were younger (*p* = 0.01); had prolonged history of fever (*p* = 0.02); had more cough and less weight (*p* = 0.01) as compared to the children managed medically
K.S. Wong (2005) [28]	Asia	81	Medical group (antibiotics with/without chest tube drainage)	Surgical intervention (VATS) In order to study the outcome of therapy and the time of surgical intervention, the patients who had undergone surgery were further subcategorized into a salvage VATS or an early VATS group.	Patients in the medical group also stayed for significantly shorter period of time as compared to the patients who had VATS. The average duration of fever was 10.6 days in the medical group vs. 12.4 days in patients who had surgery; these patients had an average stay of 23.7 and 27.4 days respectively.		Patients with an SSE (Severity Score of Empyema) of a 4 have 4.6 times chance of requiring surgery, intervention than those with SSE of < 4 in parapneumonic empyema The present data suggest that pleural pH < 7.1 and SSE > 4 are two predictors for the necessity of surgical intervention in fibrinopurulent stage of empyema. The course of conservative management was usually prolonged with a mean stay of 23.7_+8.1 days using antibiotic therapy and pleural drainage only. For patients with refractory fever, dyspneic respirations and chest pain despite adequate medical therapy and early elective VATS revealed a shorter length of hospitalization as compared to salvage VATS.
S. Sonnappa (2006) [29]	Europe	60	Percutaneous chest drain with intrapleural urokinase	VATS	No significant clinical difference in duration of hospital stay after intervention between percutaneous chest drain with intrapleural urokinase and primary VATS for the treatment of empyema in children.	There was not any adverse events directly related to the treatment in either group. However, in the urokinase group, chest drains fell out in four patients, requiring reinsertion and therefore prolonging hospital stay. Complications not directly related to the treatment included pyohemothorax post–chicken pox infection in a patient from the VATS group, lung abscess in four patients (three in the VATS group), hemolytic uremic syndrome in two patients (both in VATS group), and acute glomerulonephritis in one (urokinase group).	
B.A. Khalil (2007) [30]	Europe	38	Children were divided into four groups on the basis of the primary intervention; tube alone (group I), tube + urokinase (group II); thoracoscopy (group III) thoracotomy (group IV).		There was no difference in average length of hospital stay, post interventional stay or duration of antibiotic therapy between the groups (Thoracostomy alone, Tube + urokinase, thoracoscopy, thoracotomy).	Five children needed a secondary procedure. One child treated by tube thoracostomy alone (group I) and another child managed by thoracoscopy (group III) required thoracotomy as a secondary salvage procedure due to persistent symptoms and signs (malaise, persistent pyrexia and elevated CRP). Three children in the thoracostomy/urokinase group underwent a secondary procedure. One of these proceeded straight to thoracotomy due to unavailability of tho- racoscopic expertise whilst the other two had success- ful thoracoscopy as a secondary salvage procedure.	Amelioration of pyrexia was more rapid in children undergoing thoracotomy and there was a trend towards early normalization of CRP in the thoracotomy treatment group In this study, no differ- ences in length of hospital stay or complication rate between treatment groups were observed indicating that intrapleural urokinase is as effective as ‘‘key hole’’ thoracoscopy or thoracotomy.
J. S. Chen (2009) [31]	Asia	101	Chest tube drainage followed by thoracoscopy	Thoracoscopy as the primary treatment.	The median postoperative hospital stay was 13 days, and the median total hospital stay was 21 days. The presence of necrotizing pneumonia, preoperative chest tube drainage and preoperative ICU admission were associated with a longer postoperative hospital stay. For factors affecting total hospital stay, univariate analysis showed that preoperative empyema more than 4 days (*P* = 0.001), necrotizing pneumonia (*P* = 0.037), and preoperative chest tube drainage (*P* < 0.001) were significant factors, and that preoperative ICU admission was a borderline significant factor (*P* = 0.098). Multivariate analysis showed that that only preoperative chest tube drainage was significantly associated with a longer duration of total hospital stay (*P* < 0.001)	Preoperative ICU admission was required in 33 patients There were no mortalities. Postoperative complications developed in 10 patients (9.9%).	The mean operation time was longer in patients with preoperative chest tube drainage than in those who underwent thoracoscopy as primary treatment Prompt surgical intervention is indicated to prevent difficult operation and prolonged hospital stay, especially in patients in whom chest tube drainage is ineffective.
S.D.S. Peter (2009) [32]	North America	36	Pleural infusion of fibrinolytic solution. The fibrinolytic agent was Activase. The general antimicrobial plan for both groups consisted of clindamycin (10 mg/kg per dose) every 6 h and ceftriaxone (25 mg/kg per dose) every 12 h. If hemodynamic instability existed, then vancomycin (15 mg/kg per dose) every 6 h was added.	Video-assisted thoracoscopic debridement The general antimicrobial plan for both groups consisted of clindamycin (10 mg/kg per dose) every 6 h and ceftriaxone (25 mg/kg per dose) every 12 h. If hemodynamic instability existed then vancomycin (15 mg/kg per dose) every 6 h was added.	The outcome data showed no difference in days of hospitalization after intervention between the group trated with VATS and the group treated with fibrinolysis.	The outcome data showed no difference in days of oxygen requirement, days until afebrile, or analgesic requirements between the group trated with VATS and the group treated with fibrinolysis. Three patients (16.6%) in the fibrinolysis subsequently required VATS for definitive therapy. Two patients in the VATS group required ventilator support after therapy, one of whom continued to have progressive sepsis resulting in transient renal failure requiring temporary dialysis. No patients in the fibrinolysis group clinically worsened after initiation of therapy. No patients in either group were readmitted after discharge for ongoing or recurrent pulmonary disease.	At diagnosis, there were no differences between groups in age, weight, degree of oxygen support, white blood cell count, days of symptoms, or number of physician visits.
A. Sahin (2013) [33]	Asia	330	Initially, all patients were managed with wide-spectrum antibiotics (ampicillin/sulbactam) and were later tailored toward positive culture results. Patients were classified into five initial treatment groups: group A: thoracentesis alone group B: chest tube drainage alone; group C: fibrinolytic therapy after inadequate chest tube drainage group D: VATS following failed fibrino- lytic therapy; and group E: thoracotomy after lung entrapment and failed procedures		Patients successfully treated within group A had a shorter duration of median hospital stay of 9 days (range 5–12). Patients with VATS, group D, had a shorter postinterven- tion hospital stay of 10.0 days (range 5–11) compared with children who received fibrinolytic therapy, group C (11.0 days; range 7–17). The median hospital stay after open surgery, group E, was 10.5 days (range 6–13)	Group A: no deaths Group B: two patients died because of accompanying pneumonia and one underwent thoracotomy. Bronchopleural fistula was seen in three patients. Group C: Death followed an allergic reaction and pleural hemorrhage in one patient. One patient developed bronchopleural fistula. Group D VATS failed in five (14.28%) cases and one patient died in this group. Group E: no deaths	Thoracotomy is still needed as a last resort for cases unresponsive to chemical fibrinolysis and following failed thoracoscopy. Fibrinolytic therapy is not an alternative to surgery, especially in loculated empyemas in children. However, it should be tried in all cases of fibrinopurulent phase empyema not responding to closed chest tube drainage. This treatment increases the success of less invasive treatment. We prefer open thoracotomy or VATS for complete lung decortication, in fibrinopurulent cases, following the escalation process.
G. Grisaru-Soen (2013) [34]	Asia	47	Chest drain alone or Chest drain with fibrinolysis	VATS	The total duration of hospitalization was similar for children who underwent VATS and those who were treated conventionally (14.42 days vs. 14.46 days, respectively), and there was no difference in the mean hospital stay between the 2 participating hospitals (14.52 days in Dana vs. 14.78 days in Safra).	No difference in complications between the two approaches. There was no empyema-associated mortality. Most children in the empyema group (*n* = 45) were treated in the pediatric intensive care unit for at least 24 h and all of them needed a chest drain, a chest drain with fibrinolysis, or decortication with video-assisted thoracoscopic sur- gery (VATS)	There was no difference in outcome as reflected by the length of stay and complications between the operative (VATS) treatment group and nonoperative treatment group, suggesting that the former does not result in shorter hospital stay or fewer complications compared with the latter. A prospective study is warranted to compare the effect of VATS with chest drain and fibrinolysis on the outcome of pediatric patients with empyema.
A. Budusan (2013) [35]	Europe	16	antibiotic + chest drain	Open thoracotomy and decortications	Length of hospital stay was longer for children who had open thoracotomy with decortication rather than children terated with only antibiotic and chest drain, because they were previously treated with chest-drain tubes, with poor outcome or results. The median hospital stay was 15.5 days in group I (antibiotic + chest drain) and 24.25 days in group II (thoracotomy and decortication).	In our group of 16 pediatric patients, we had no mortality. Morbidity referred to presence of bronchopleural fistulas which were identified by the presence of air in pleural space (hydropneumothorax), and were treated using only underwater sealing drainage (4 cases in group II) or by closing fistula during thoracotomy (1 case in group II) and one case of lung abscess in group I, treated only with antibiotics, with good outcome.	
T. Alar (2013) [36]	Asia	38	Medical treatment	Surgical treatment (Decortication Decortication + cavity obliteration Decortication + wedge resection Decortication + segmentectomy Decortication + lobectomy)	An analysis of the total length of hospitalization of the two different groups showed that those treated medically had significantly shorter inpatient duration than the surgically treated group (*p* = 0.010). However, evaluation of the postoperative length of hospitalization revealed that the surgically treated patients stayed significantly shorter periods of time in the hospital than those who were treated medically (*p* < 0.001).	A bronchopleural fistula (BPF) was found in eight (21%) patients and all were in the surgical treatment group.	Clinicians should use the surgical treatment option if a BPF is present. Otherwise, they can wait patiently for the cavitary lesions to regress and postpone the open surgical treatment option until the recovery period.
Rajesh Kumar Singh et al. (2020) [37]	Asia	13	Primary VATS Conservative (Only antibiotics) ICD ICD followed by VATS Conservative followed by VATS		Most of the patients were treated by VATS and compared to pleurical drenage group the duration of stay was lesser for VATS but this was not statistically significant (*p* > 0.05). The mean average length of hospital stay was comparable between those children who underwent primary VATS versus other modes of treatment.	Out of 13 cases managed there were no deaths.	

### Mortality

Mortality data was reported in 100 out of 127 studies. Two studies did not mention mortality in outcome (2/127). Among the studies reporting mortality (25/127), the mortality rate was below the 5% of the sample size (21/25) and under 15% in the rest of cases (4/25). Overall, 78 deaths occurred among the 10,896 children (0.7%) included in the review.

Focusing on the region, mortality was predominantly in developing countries (19/25); the majority were conducted in Asia, while the highest percentages of mortality related to sample size (up to 10% of the sample size) were recorded in studies conducted in Africa.

## Discussion

This scoping review mapped the existing literature on pediatric empyema published over the past 23 years. To the best of our knowledge, this represents the most extensive description of current knowledge in the topic. Overall, we have found that evidence on the topic is still inconclusive and difficult to translate into rigorous guidelines. Aetiologies are frequently reported, with a probable role of molecular assays in improving microbe detection. As expected, *S. pneumoniae*, Group A *Streptococcus* and *S. aureus* are the most frequent pathogens. However, gaps remain: the serotypes of *S. pneumoniae* are rarely reported, as well as children’ vaccination status. Therefore, the real burden of *S. pneumoniae* serotype 3 in empyema in vaccinated children remains unclear, as current vaccines may offer/provide lower protection against it [38]. Studies have in fact described vaccine breakthrough cases of serotype 3 complicated pneumonia in vaccinated children [38]. Additionally, the distinction between methicillin-susceptible *S. aureus* (MSSA) and methicillin-resistant *S. aureus* (MRSA) is seldom reported, even though their pathogenicity might differ. Finally, *M. pneumoniae* was rarely identified as a leading pathogen.

Currently, there are no rigorous guidelines concerning antibiotic treatment for pediatric empyema, and the literature still appears lacking solid scientific evidence, which is why pediatricians relies mainly on expert opinions. It is common to start with empirical broad-spectrum antibiotics which can be switched to narrow-spectrum following examination culture and susceptibility testing also considering local antibiotic resistance. However, while this is what is usually suggested, in routine practice there is confusion in terms of number of drugs, routes and length of antibiotic therapies, as well as optimal surgical approaches.

In general, we observed that the most common performed surgical treatment was pleural drainage, while the less performed one was open surgery, reserved for the most severe cases. Video-assisted thoracoscopic surgery (VATS) and fibrinolysis were performed almost equally. However, the studies included patients with diverse characteristics and employed different criteria for inclusion. Additionally, the causative organisms were often unknown, making it difficult to translate these findings directly into routine clinical practice-. Specifically, a recent ongoing systematic review and meta-analysis, which continuously updates its findings, compared various treatment approaches for pediatric empyema management. This review revealed that therapies such as fibrinolytic therapy, (VATS), and thoracotomy were linked to shorter hospital stays compared to chest tube drainage alone [39]. However, is it important to acknowledge that assessing hospital length of stay can be subjective, potentially limiting the generalizability of these findings. Notably, short- and long-term morbidity rates were similar across different treatment options, and mortality rates were low across all interventions, consistent with the outcomes observed in our study. In addition, inclusion criteria for the diagnosis of empyema varied. Some studies included biochemical findings in the pleural fluid, while others considered loculations on imaging. However, interpreting pleural fluid loculations can be subjective, and lung ultrasound might be a more reliable imaging tool for this purpose [40], which however has only recently been significantly implemented in pediatric practice [41]. Small studies conducted by expert pediatricians in the field found that lung ultrasound seems to be very sensitive in predicting empyema, and may guide therapeutic choices. However, there are currently no trials to evaluate whether children diagnosed with lung ultrasound and given to different pharmacological arms may benefit from one intervention over another.

Importantly, another limitation in the interpretation of available literature is the absence of clinical trials comparing antibiotic therapies while having a fixed surgical intervention, nor trials comparing surgical approaches while having fixed antibiotic therapies. As such, it is challenging to understand the impact of each drug, or surgical intervention, on the patient’s improvement. Our review identified that children often undergo multiple antibiotic treatments due to an apparent lack of clinical response to pharmacological treatment. However, in many cases, it is possible that persistence of fever may not to be attributed to the lack of pharmacological efficacy but could simply be due to the persistence of inflammation. As such, multiple antibiotics are frequently administered, whereas a single effective antibiotic paired with a single effective surgical approach may suffice. The absence of bacteria-specific trials makes it difficult to provide clear treatment recommendations. Also, a recent brief review from the European Society of Pediatric Infectious Disease provides reasonable options rather than defined indications [42], and also five of the major international societies have similar but still different recommendations [43–47]. Of note, only a few studies have described the role of newer drugs with optimal lung penetration such as linezolid, yet with no trials available.

Importantly, despite the mentioned limitations of the available literature and uncertainties about optimal management, mortality in children with empyema is overall low. However, significant differences have been highlighted between high and low-to-middle income countries. Such differences may be multifactorial, both associated with availability of healthcare resources like intensive care units and surgery, but also pathogen- (e.g., multidrug resistance in specific countries) and host- (e.g., malnutrition, genetics, delay in diagnosis due to socioeconomic issues) related factors. Such differences would need more studies to better understand this point and reduce inequalities in children’s outcomes.

## Conclusions

Despite an increase in pediatric empyema research over the past two decades, a significant gap exists in high quality clinical trials. This hinders to fully understand the disease and the optimal surgical approach. While some existing surgical trials suggest potential benefits for fibrinolysis in terms of safety, costs and success rates, the best antibiotic regimen remain unclear. Future well-designed trials should aim to investigate different antibiotics (including newer ones with optimal lung penetration) accompanied by fixed surgical approaches, as well as different surgical interventions accompanied by optimal antibiotic therapies.

### Electronic supplementary material

Below is the link to the electronic supplementary material.

Supplementary Material 1

## Data Availability

available upon request to the corresponding author.

## Abbreviations

CAP: Community-acquired pneumonia
PCV: Pneumococcal conjugate vaccine
CT: Computed tomography
MSSA: Methicillin-susceptible S. aureus
MRSA: methicillin-resistant S. aureus
VATS: Video-assisted thoracoscopic surgery
